# Supplementary material for: WordCommentsAnalyzer: A windows software tool for qualitative research
Source: F1000Res. 2018 Sep 4;7:536. Originally published 2018 May 3. [Version 2] doi: 10.12688/f1000research.14819.2 (PMC6137411; doi:10.12688/f1000research.14819.2)
Supplement: Supplementary file 3 [file f1000research-7-17614-s0002.tgz › b2a79496-4ada-47c4-9682-a99ce867836d.zip!/Eight Iranian Health Journals/Journal of Education and Community Health_7.docx]

© Journal of Education and Community Health. This work is licensed under CC BY-NC 4.0 (https://creativecommons.org/licenses/by-nc/4.0/).

Volume & Issue: دوره 3 - شماره 2

Title: تعیین کننده‌های شناختی-رفتاری بهداشت دهان و دندان در دانشجویان: کاربردی از مدل ارتقاء سلامت پندر

http://jech.umsha.ac.ir/article-1-215-fa.html

سابقه و هدف: شناسایی عوامل مؤثر بر رفتارهای بهداشت دهان و دندان در دانشجویان می‌تواند نقش مهمی در ارتقاء سلامت دهان و دندان آنها داشته باشد. لذا مطالعه حاضر با هدف بررسی تعیین کننده‌های شناختی-رفتاری بهداشت دهان و دندان در دانشجویان انجام گرفته است. مواد و روش‌ها: در این مطالعه مقطعی، 251 نفر از دانشجویان دانشگاه‌های آزاد، پیام نور و علوم پزشکی سراب در سال 1395 به روش نمونه گیری تصادفی ساده انتخاب گردیدند. ابزار جمع‌آوری اطلاعات شامل پرسشنامه استاندارد مشتمل بر سه بخش متغیرهای جمعیت شناختی، سازه‌های مدل ارتقاء سلامت پندر و رفتارهای بهداشت دهان و دندان بود. داده‌ها با نسخه 21 نرم افزار SPSS و آزمون‌های تی‌مستقل، آنالیز واریانس یک‌طرفه، همبستگی پیرسون و آنالیز رگرسیون خطی تحلیل گردید. یافته‌ها: میانگین سنی شرکت کنندگان در پژوهش 1.67±21.9 بود. رفتار بهداشت دهان و دندان با تمام سازه‌های مدل ارتقاء سلامت پندر به جز تأثیرگذارنده‌های موقعیتی (0.048=r) همبستگی آماری معنی‌داری داشت. سازه‌های مدل ارتقاء سلامت پندر قادر به پیش بینی 43.6 درصد رفتار بهداشت دهان و دندان و 26.4 درصد تعهد به عمل بودند. خودکارآمدی درک شده قویترین پیش بینی کننده رفتار (0.342=β) و موانع درک شده قویترین پیش‌بینی کننده تعهد به عمل (0.217-=β) بود. نتیجه‌گیری: طبق یافته‌ها، خودکارآمدی قویترین پیش‌بینی کننده رفتار بهداشت دهان و دندان و موانع درک شده قویترین پیش‌بینی کننده تعهد به طرح عمل بود. به طور کلی براساس یافته‌های مطالعه حاضر، مدل ارتقاء سلامت پندر می‌تواند به عنوان یک چارچوب مناسب در شناسایی عوامل و طراحی مداخلات مؤثر جهت بهبود رفتارهای بهداشت دهان و دندان در دانشجویان استفاده شود.

Title: بررسی ارتباط بین ادراک بیماری و تبعیت از رژیم درمانی در بیماران مبتلا به پرفشاری خون

http://jech.umsha.ac.ir/article-1-219-fa.html

سابقه و هدف: بیماری پرفشاری خون به عنوان یک مشکل مهم بهداشت عمومی در سراسر دنیا شناخته شده است. ادراک بیماری بازنمایی شناختی سازمان یافته بیمار از بیماری خود می ­باشد و می‌تواند تبعیت از درمان بیمار را تحت تأثیر قرار دهد. لذا، این مطالعه با هدف بررسی ارتباط بین ابعاد مختلف ادراک بیماری و تبعیت از رژیم درمانی در بیماران مبتلا به پرفشاری خون انجام گردید. مواد و روش‌ها: در این مطالعه­ مقطعی، 140 بیمار مبتلا به پرفشاری خون مراجعه کننده به بیمارستان­ های آموزشی شهر قم در سال 1395 به روش نمونه­ گیری آسان انتخاب شدند. ابزار گردآوری داده ­ها پرسشنامه اطلاعات جمعیت‌شناختی و پرسش­نامه­ فرم کوتاه ادراک بیماری و مقیاس تبعیت به درمان‌های فشار‌خون بالا بود. داده‌ها با استفاده از آمار توصیفی، ضریب همبستگی پیرسون و تحلیل رگرسیون چند متغیره در محیط نرم افزار آماری SPSS-16 تجزیه و تحلیل گردید. یافته‌ها: میانگین سنی بیماران 11.21±60.40 بود و 57 درصد از آنان را زنان تشکیل می­دادند. 35 درصد بیماران تبعیت از درمان بالاتر از میانگین داشتند. بر اساس نتایج تحلیل رگرسیون چند متغیره کنترل شخصی و قابلیت درک و فهم بیماری به ترتیب با ضریب بتای 25 درصد و 22 درصد مهمترین پیش بینی کننده‌های تبعیت از رژیم درمانی بودند. نتیجه‌گیری: با توجه به نتایج مطالعه، تقویت ادراک بیماری به خصوص دو جزء قابلیت درک و فهم بیماری و کنترل شخصی به منظور افزایش تبعیت از درمان بیماران، به عنوان یک راهبرد مهم در مداخله ­های آموزشی ضروری به نظر می ­رسد.

Title: میزان شیوع پاپ اسمیرهای غیرطبیعی در زنان مراجعه کننده به مراکز بهداشتی درمانی وابسته به علوم پزشکی همدان در طی سال‌های 1390- 1394

http://jech.umsha.ac.ir/article-1-221-fa.html

سابقه و هدف: سرطان دهانه رحم یکی از مهمترین بیماری‌های دستگاه تناسلی زنان است. مطالعه حاضر با هدف بررسی شیوع پاپ اسمیرهای غیرطبیعی در زنان مراجعه کننده به مراکز بهداشتی درمانی وابسته به علوم پزشکی همدان انجام گردید. مواد و روش‌ها: در طی یک مطالعه گذشته نگر 36046 نتیجه پاپ اسمیر استخراج شده از پرونده زنان مراجعه کننده به مراکز بهداشتی درمانی دولتی وابسته به دانشگاه علوم پزشکی همدان و بیمارستان زنان فاطمیه شهر همدان بین سال‌های 1390-1394 بررسی شد که  605 مورد غیرطبیعی، جمع آوری و مورد بررسی و مقایسه قرار گرفت. ابزار جمع ­آوری اطلاعات، چک لیست بود. داده‌ها با نسخه 21 نرم افزار SPSS و با استفاده از روش‌های آمار توصیفی تحلیل گردید. یافته ­ها: 30.4 درصد از زنان دارای نتایج پاپ اسمیر غیرطبیعی در محدوده سنی 45-36 سال بودند و 48.6 درصد از آنان سابقه 1 تا 3 بارداری را گزارش کرده بودند. از کل 36046 پرونده مورد بررسی، 605 مورد پاپ اسمیر غیرطبیعی دیده شد. بیشترین فراوانی پاپ اسمیر غیر طبیعی مربوط به ASCOUS با 78 درصد و کمترین فراوانی مربوط به LSIL با 1 درصد بود. بین نتایج غیرطبیعی پاپ اسمیر با سن افراد ارتباط مستقیم و معنی‌دار آماری مشاهده شد (P=0.037). نتیجه‌گیری: شیوع پاپ اسمیر غیرطبیعی در استان همدان، 1.67 درصد گزارش شد. ریسک سرطان‌های بدخیم سرویکس و سرطان‌های مهاجم با بالارفتن سن افزایش می‌یافت، بنابراین غربالگری و انجام تست پاپ اسمیر بخصوص از سن 35 به بالا توصیه می‌شود.

Title: بررسی اثربخشی آموزش غنی‌سازی روابط بر ارتقاء کیفیت زندگی و رضایت زناشویی دانشجویان متأهل

http://jech.umsha.ac.ir/article-1-214-fa.html

سابقه و هدف: توجه به زندگی زناشویی و تلاش جهت افزایش مؤلفه‌های مرتبط با زندگی زناشویی رشد روزافزونی را به خود گرفته است، چرا که نمی‌توان جامعه سالم بدون خانواده سالم را متصور بود. بر همین اساس هدف این پژوهش بررسی اثربخشی آموزش غنی‌سازی روابط بر ارتقاء کیفیت زندگی و رضایت زناشویی دانشجویان متأهل دانشگاه خمینی‌ شهر بود. مواد و روش‌ها: پژوهش حاضر یک کارآزمایی بالینی با طرح پیش‌آزمون-پس‌آزمون همراه با گروه کنترل بود. جامعه آماری پژوهش حاضر را تمام دانشجویان زن متأهل دانشگاه خمینی شهر اصفهان تشکیل می‌دادند. به منظور انجام پژوهش، تعداد 32 نفر از دانشجویان متأهل زن دانشگاه آزاد خمینی شهر به شیوه نمونه گیری در دسترس و هدفمند انتخاب و به صورت تصادفی به دو گروه آزمون و کنترل تقسیم شدند. گروه آزمون در دوره آموزش غنی‌سازی روابط طی 7 جلسه 90 دقیقه‌ای شرکت کردند و در گروه کنترل هیچ مداخله‌ای صورت نگرفت. ابزار پژوهش شامل مقیاس کیفیت زندگی و رضایت زناشویی بود. برای تحلیل داده‌ها از تحلیل کوواریانس با استفاده از بسته نرم افزاری SPSS-20 استفاده شد. یافته‌ها: تحلیل نتایج بیانگر این بود که بین میانگین نمرات مقیاس کیفیت زندگی و رضایت زناشویی شرکت‌کنندگان برحسب عضویت گروهی (دو گروه آزمون و کنترل) پس از تعدیل تأثیر متغیر پیش‌آزمون تفاوت معنی‌داری وجود دارد (0.001P<). نتیجه‌گیری: با توجه به تأثیر مثبت آموزش غنی سازی روابط بر کیفیت زندگی و رضایت زناشویی می‌توان از این روش جهت افزایش کیفیت زندگی و رضایت زناشویی و در نتیجه آن رضایت از زندگی زوجین بهره برد.

Title: تأثیر آموزش برنامه ورزشی بر تغییر رفتارهای پیشگیرانه کمردرد در زنان باردار مراجعه کننده به مراکز سلامت جامعه کرج

http://jech.umsha.ac.ir/article-1-209-fa.html

سابقه و هدف: کمردرد یکی از شایع‌ترین علائم وابسته به بارداری است، لذا این پژوهش با هدف تعیین تأثیر آموزش برنامه ورزشی بر تغییر رفتارهای پیشگیرانه کمردرد در زنان باردار انجام گردید. مواد و روش‌ها: پژوهش حاضر یک مطالعه نیمه تجربی از نوع پیش‌آزمون -پس‌آزمون همراه با گروه کنترل بود که جامعه آماری آن 120 زن باردار مراجعه‌کننده به مراکز سلامت شهر کرج بودند که به‌صورت تصادفی به دو گروه مداخله (60 نفر) و کنترل (60 نفر) تقسیم شدند. ابزار گردآوری داده‌ها شامل مشخصات جمعیت‌شناختی، مقیاس دیداری درد، پرسشنامه سنجش آگاهی، نگرش و رفتارهای پیشگیرانه از کمردرد بود. داده‌ها با استفاده از نسخه 21 نرم‌افزار SPSS و آزمون‌های تی‌زوجی، من ویتنی و آزمون توکی تحلیل گردید. یافته‌ها: بین میانگین نمرات آگاهی، نگرش و رفتارهای پیشگیرانه از کمردرد دوران بارداری و همچنین شدت درد بر اثر کمردرد در سه‌ماهه سوم و در گروه مداخله قبل و بعد از آموزش اختلاف معنی‌داری مشاهده شد (0.001P<). همچنین اختلاف معنی‌داری در مؤلفه‌های ذکرشده در دو گروه آزمون و کنترل وجود داشت (0.001P<).  نتیجه‌گیری: این مطالعه نشان داد که مداخله آموزشی می تواند باعث بهبود آگاهی، نگرش و رفتار زنان باردار در رابطه با رفتار پیشگیرانه از کمردرد گردد.

Title: تأثیر آموزش ترکیبی بر ارتقاء رفتارهای تغذیه ای دانش آموزان دختر

http://jech.umsha.ac.ir/article-1-87-fa.html

سابقه و هدف: سوءتغذیه یکی از مهم‌ترین معضلات بهداشتی دنیای امروز است که میلیون‌ها کودک را در سراسر جهان تحت تأثیر قرار می ­دهد. بنابراین این مطالعه با هدف تعیین تأثیر برنامه آموزشی بر رفتار‌های تغذیه‌ای دانش‌آموزان دختر 8 تا 10 ساله شهر چابهار انجام شد. مواد و روش‌ها: پژوهش حاضر یک مطالعه نیمه تجربی از نوع قبل و بعد بود. جامعه آماری پژوهش حاضر را تمام دانش­آموز دختر 8 تا 10 ساله شهر چابهار در سال 1393 تشکیل می‌دادند. به‌منظور انجام پژوهش، تعداد 65 نفر از دانش آموزان دختر به شیوه نمونه‌گیری تصادفی ساده انتخاب و وارد مطالعه شدند. ابزار گرد­آوری اطلاعات شامل اطلاعات جمعیت شناختی و پرسشنامه­های آگاهی، نگرش و عملکرد بود که قبل از آموزش، بلافاصله و دو ماه بعد از آخرین مداخله­ی آموزشی تکمیل گردید. مداخله­ آموزشی با استفاده از آموزش ترکیبی (سخنرانی، چندرسانه‌ای) در 3 جلسه­ 30 دقیقه­ای انجام گردید. برای تحلیل داده‌ها از آزمون اندازه‌گیری‌های تکراری و کای دو با استفاده از بسته نرم‌افزاری SPSS-16 استفاده شد.  یافته­ ها: نتایج نشان داد که بین میانگین نمره­ آگاهی دانش‌آموزان قبل از آموزش با بلافاصله و دو ماه بعد اجرای برنامه مداخله‌ای تفاوت معنی‌داری وجود دارد (0.05>P). ولیکن بین میانگین نمرات نگرش و عملکرد قبل و بعد از آموزش تفاوت معنی­ داری مشاهده نشد (0.05 نتیجه­ گیری: نتایج نشان داد که آموزش ترکیبی به علت بکار گرفته شدن حواس بیشتر در افزایش آگاهی مؤثر است اما درزمینهٔ تغییر نگرش و عملکرد نیازمند بررسی­ های بیشتر و انجام مداخلات در مدت زمان طولانی­ تر می­ باشد.

Title: مقایسه تأثیر دو برنامه تمرین تناوبی با شدت بالا (HIIT) به مدت دوهفته بر اکسیداسیون چربی، درصدچربی بدن و VO2max در مردان جوان دارای اضافه وزن

http://jech.umsha.ac.ir/article-1-108-fa.html

سابقه و هدف: پژوهش حاضر با هدف مقایسه تأثیر دو برنامه تمرین تناوبی با شدت بالا)  (High Intensity Interval Training (HIIT) بر اکسیداسیون چربی، درصد چربی و VO2max در مردان جوان دارای اضافه وزن بود. مواد و روش ­ها: 16 مرد جوان غیرفعال دارای اضافه وزن به ­صورت داوطلبانه در این پژوهش نیمه تجربی شرکت و به­ طور تصادفی به دو گروه HIIT-1 و گروه HIIT-2 تقسیم شدند. گروه HIIT-1 به مدت دو هفته و سه جلسه در هفته پروتکل تمرینیHIIT  (4 وهله فعالیت یک دقیقه‌ای با 4 دقیقه استراحت) و گروه HIIT-2 (4 وهله فعالیت 30 ثانیه‌ای با 2 دقیقه استراحت) را با حداکثر سرعت در یک ناحیه 20 متری انجام دادند. گازهای تنفسی یک روز قبل و بعد از تمرین، به­ صورت ناشتا برای انجام تجزیه و تحلیل­ های آزمایشگاهی جمع­ آوری شد. داده­ ها در نرم افزار SPSS-18 و با استفاده از آزمون ­های­ آماری تی مستقل و تی­ وابسته تحلیل گردید. یافته‌ها: نتایج نشان داد که 6 جلسه اجرای HIIT موجب افزایش 43 درصدی اکسیداسیون چربی به هنگام تست بروس در گروه HIIT-1 گردید که این تغییرات در مقایسه با گروه HIIT-2 از نظر آماری نیز معنی­ دار بود (0/05P<). تغییرات وزن و درصد چربی بدن تنها در گروه HIIT-1 معنی­ دار بود (0/05P<). همچنین، افزایش معنی­ دار 19/5 درصدی VO2max در گروه HIIT-1 و افزایش معنی­ داری 4 درصدی در گروه HIIT-2 مشاهده گردید (0/05P<). نتیجه‌گیری: با توجه به نتایج پژوهش حاضر، اجرای 6 جلسه HIIT منجر به بهبود نسبی اکسیداسیون چربی، VO2max، کاهش درصد چربی بدن و وزن می‌شود. البته نقش پروتکل تمرینی گروهHIIT-1  بیشتر بوده و می ­تواند علاوه بر کاهش مؤثر چربی بدن و افزایش آمادگی هوازی، از نظر تأثیر زمانی یک پروتکل کارآمد برای ارتقاء سلامتی و کاهش وزن باشد.

Title: اثربخشی برنامه آموزشی مبتنی بر نظریه رفتار برنامه‌ریزی شده در پیشگیری و کاهش مصرف دخانیات در دانشجویان

http://jech.umsha.ac.ir/article-1-212-fa.html

سابقه و هدف: مصرف دخانیات در بین جوانان در سال‌های اخیر در حال افزایش می­باشد. بنابراین مطالعه حاضر با هدف بررسی تأثیر برنامه آموزشی مبتنی بر تئوری رفتار برنامه ریزی شده در پیشگیری و کاهش مصرف دخانیات در دانشجویان دانشگاه علوم پزشکی لرستان انجام گردید. مواد و روش‌ها: در این مطالعه نیمه‌تجربی، 126 نفر از دانشجویان پسر دانشگاه علوم پزشکی لرستان به‌صورت تصادفی به دو گروه آزمون و کنترل تقسیم ‌شده بودند. ابزار گردآوری اطلاعات شامل پرسشنامه مبتنی بر نظریه رفتار برنامه‌ریزی و اطلاعات جمعیت‌شناختی آزمودنی‌ها بود. برنامه مداخله‌ای شامل 4 جلسه آموزشی بود که در مدت زمان 1 ماه برای شرکت‌کنندگان گروه آزمون به مرحله اجرا درآمد. دو گروه آزمون و کنترل سه ماه پس از مداخله مورد پیگیری قرار گرفتند. داده‌ها با استفاده از نرم‌افزارهای SPSS-18 و با بکارگیری آزمون کای‌اسکوئر، تی‌مستقل، تی‌زوجی و مک نمار مورد تجزیه ‌و تحلیل  قرار گرفتند.   یافته‌ها: اختلاف­ های معنی‌داری برای میانگین نمرات نگرش، هنجارهای انتزاعی و قصد رفتاری بین گروه‌های آزمون و کنترل بعد از اجرای برنامه مداخله‌ای مشاهده گردید (0/05P<). همچنین یافته­ های مطالعه حاضر نشان داد که اختلاف معنی­ داری در خصوص مصرف سیگار و قلیان در بین دانشجویان گروه آزمون و کنترل بعد از اجرای مداخله آموزشی وجود دارد (0/05P<). نتیجه‌گیری: اجرای جلسات آموزشی مبتنی بر نظریه رفتار برنامه­ ریزی شده موجب پیشگیری و کاهش مصرف دخانیات در دانشجویان می­ گردد
